# 0355. Optimal chest compression technique for pediatric arrest victims

**DOI:** 10.1186/2197-425X-2-S1-P21

**Published:** 2014-09-26

**Authors:** MJ Kim, YS Park

**Affiliations:** Yonsei University College of Medicine, Department of Emergency Medicine, Seoul, Korea, Republic of

## Introduction

In pediatric cardiopulmonary resuscitation (CPR), high-quality chest compressions (CCs) are essential to successful resuscitation, although most pediatric arrests are caused by respiratory arrest. Therefore, current guidelines emphasize deeper and faster CCs, with complete chest recoil between contractions.^1, 2^ These guidelines further indicate that rescuers may use a one-hand or two-hand technique for pediatric compressions, thereby allowing rescuers to adapt the technique to the victim's size and rescuer's strength. However, no previous studies have examined the quality of CCs, including the use of deeper and faster CCs with complete chest recoil emphasized in the guidelines. Additionally, no study has assessed compression quality of the one-hand technique comparing the right and left hands.

## Objectives

The aim of this study was to assess the quality of CCs performed by inexperienced rescuers using three different techniques: two-hand (2H), right one-hand (R1), and left one-hand (L1).

## Methods

We performed a prospective, randomized, crossover study in a simulated 6-year-old pediatric manikin model. Each participant performed 2-minute continuous CCs, using three different techniques (2H, R1, and L1). CC quality data, including compression rate, compression depth, and leaning, were collected and analyzed. To examine trends in CC performance over time, each 2-minute period was divided into six consecutive 20-second epochs.

## Results

The 36 participants completed 108 two-minute trials, consisting of a total of 25,030 CCs. The mean compression rates (95% confidence interval [CI]) were as follows: 2H1, 116.8 compressions/minute (111.7-121.9); L1, 115.0 compressions/minute (109.9-120.1); and R1, 115.5 compressions/minute (110.4-120.6) (p=0.565). The mean compression depth for 2H was 38.7 mm (37.1-40.2), which was higher than for L1 (36.3 mm [34.8-37.9]) or R1 (35.4 mm [33.9-37.0]) (p< 0.001). The leaning rate was higher with 2H than L1 or R1 (p=0.021). Chest compression depth per 20-second epoch declined over time, regardless of the technique (p< 0.001). The pattern of compression depth change over time was similar for all techniques (p>0.999).

## Conclusions

For pediatric cardiopulmonary resuscitation by inexperienced rescuers, the two hand CC technique has the advantage of producing deeper compressions than the one-hand technique, but it is accompanied by more frequent leaning. For the one-hand techniques, the right and left hand produced CCs of similar quality.
